# Consumption of Meats and Fish in Poland during the COVID-19 Lockdown Period

**DOI:** 10.3390/nu16091318

**Published:** 2024-04-27

**Authors:** Dominika Szajnoga, Helena Perenc, Grzegorz K. Jakubiak, Grzegorz Cieślar, Małgorzata Ćwieląg-Drabek

**Affiliations:** 1 Student Research Group, Department of Environmental Health, Faculty of Public Health in Bytom, Medical University of Silesia, Piekarska 18 St., 41-902 Bytom, Poland; szajnogadominika@gmail.com; 2 Student Research Group, Department and Clinic of Internal Medicine, Angiology, and Physical Medicine, Faculty of Medical Sciences in Zabrze, Medical University of Silesia, Batorego 15 St., 41-902 Bytom, Poland; perenc.helena@gmail.com; 3 Department and Clinic of Internal Medicine, Angiology, and Physical Medicine, Faculty of Medical Sciences in Zabrze, Medical University of Silesia, Batorego 15 St., 41-902 Bytom, Poland; cieslar1@tlen.pl; 4 Department of Environmental Health Risk Factors, Faculty of Public Health in Bytom, Medical University of Silesia, Piekarska 18 St., 41-902 Bytom, Poland; mdrabek@sum.edu.pl

**Keywords:** COVID-19, lockdown, meat consumption, fish consumption

## Abstract

The COVID-19 pandemic and related restrictions have significantly impacted the quality of life of society in many countries in various aspects. The purpose of this study was to examine how the COVID-19 pandemic affected the consumption of meat and fish in society in Poland as well as the factors that determined these changes. The cross-sectional study was conducted using an original online questionnaire between 8th and 18th of April 2020. The subjects were selected randomly. The target population were adults living in Poland. The inclusion criterion for the study was the age of at least 18 years and consent to selfless participation in the study. Communication via social media such as Facebook or Instagram was used to enroll more participants from different socio-demographic subgroups. The questionnaire was divided into two parts—the first part contained questions about sex, age, body mass, body height, residence, level of education, and occupation, and the second part consisted of specific questions about the consumption frequency of different kinds of meat during the COVID-19 lockdown period (in comparison to the time before the pandemic). A total of 3888 people took part in the study including 84.54% women, with an average age of 30.17 ± 9.22 years. The frequency of poultry consumption increased, while for pork, beef, ham and other meat products as well as fish and seafood it declined. The factors that influenced the change in dietary patterns during the lockdown related to the COVID-19 pandemic included age, body mass index, gender, place of residence, and type of work performed. However, no relationship was found between the change in meat and fish consumption during the pandemic and the level of education and form of employment during the pandemic.

## 1. Introduction

COVID-19 is an infectious disease whose etiological factor is the SARS-CoV-2 coronavirus [1]. The first case of COVID-19 was diagnosed in December 2019 in Wuhan, China [2]. Although the greatest threat in the course of COVID-19 is pneumonia complicated by respiratory failure, the symptoms and complications of COVID-19 may affect various organ systems [3,4,5,6]. Over the following years, the COVID-19 pandemic was a leading public health problem around the world, and the efforts of researchers focused on the development of treatment methods [7,8,9,10] and the identification of factors associated with its severe course [11,12,13,14,15]. A major breakthrough in the fight against COVID-19 was the introduction of vaccinations [16].

Although the threat from COVID-19 is currently much lower, it should be noted that the COVID-19 pandemic considerably influenced the everyday life of different societies. Social campaigns and new legislation shaped the reality of online schooling, remote work, the interruption of international transport, and closed public facilities, as well as frequent disinfection, mandatory masks, and isolation. Various changes were made in almost every aspect of human functioning including nutrition.

For a couple of decades, understanding the determinants of dietary habits was the purpose of many studies. Kurt Lewin described dietary behaviors as a complex issue, influenced by cultural, social, and psychological factors [17]. Accordingly, limitations due to the COVID-19 pandemic played a role in this process. Specific guidelines targeting shopping resulted in buying increased amounts of food in order to minimize the frequency and the time spent in grocery stores. Food service also underwent changes. The lack of being able to consume food in restaurants made take-out and delivery services develop rapidly. Changes in dietary patterns were not only the result of individual consumer choices, but also problems in production and disrupted supply chains. The availability of food including meat changed because of the COVID-19 pandemic in many countries, and the production of meat decreased as a result of COVID-19 infections affecting employees of the meat industry and disrupted transportation. More difficult access and the tendency to buy an abundance of food at the beginning of the pandemic influenced the rise in meat prices [18].

According to the report prepared by the Responsible Business Forum (a Polish non-governmental organization), 84% of Polish people searched for news regarding the coronavirus several times a day at the end of March 2020, which directly contributed to the perception of the pandemic as having an influence on everyone’s daily lives [19]. This emotional aspect of human functioning should not be overlooked while examining dietary behaviors. The beginning of the COVID-19 pandemic was an unfortunate time of great emotional pressure that resulted in a more common occurrence of mental disorders such as depression or anxiety [20,21,22]. Sygit-Kowalkowska has stated that food choices and patterns of consumption are strictly connected to the emotional state of an individual [23].

A better understanding of the dietary behaviors of Polish people during the pandemic may be crucial for the adequate planning of further actions. The excess consumption of meat is connected to the increased frequency of multiple diseases [24]. Diet, as a modifiable element of lifestyle, seems to play a significant role in the course of coronavirus infection. An overabundance of accessible highly-processed, energy-dense, and low-nutrient food may be a reason for obesity, which is a significant risk factor for hospitalization and death due to COVID-19. It has been estimated that as many as 30% of hospitalizations of COVID-19 patients in the United Kingdom may have been the result of excessive body weight [25]. Furthermore, over 60% of hospitalizations of COVID-19 patients in the USA were directly caused by three disease entities connected to diet and other lifestyle elements: arterial hypertension, type 2 diabetes, and heart failure [26]. Due to these factors, a decline in the consumption of meat, especially those that are highly processed, would be beneficial. On the other hand, a lower meat consumption might reflect a lack of food safety [27]. Many people may face problems in fulfilling their dietary needs during a pandemic. Taking into account the above-mentioned aspects, examining the factors that may influence the dietary habits of the population is important, and one of these factors may be restrictions related to the COVID-19 pandemic.

Climate change is also a risk factor for the spread of zoonoses, which means that the population might encounter the problem of future pandemics, therefore, a deeper knowledge regarding dietary habits in such circumstances could be useful [28].

The purpose of this study was to examine how the consumption of individual types of meat and fish changed during the early phase of the COVID-19 pandemic in Poland including expanding knowledge about the factors influencing these changes.

## 2. Materials and Methods

### 2.1. Research Instrument

The study was conducted using an original online questionnaire between the 8th and 18th of April 2020. The subjects were selected randomly. The survey was conducted online using random sampling selection. The target population were adults living in Poland. Communication via social media such as Facebook or Instagram was used to enroll more participants from different socio-demographic subgroups. The questionnaire was divided into two parts—the first contained questions about sex, age, body mass, body height, residence, level of education, and occupation, and second part consisted of specific questions about the consumption frequency of different kinds of meat during the COVID-19 lockdown period (in comparison to the time before the pandemic). Participants could choose one of the answers describing changes in consumption as follows: “much less frequently”; “slightly less frequently”; “the same as before”; “slightly more frequently”; or “much more frequently”.

Participants who were not working during the lockdown period due to temporary circumstances (maternity leave, sick leave, temporary benefits) as well as students, the unemployed, and people who lost their jobs because of the pandemic were included in the “did not work” group.

People who performed jobs in changing places (e.g., professional drivers, delegates), farmers, and self-employed people were included in the “non-remote work” group.

### 2.2. Assessment of Nutritional Status

Nutritional status was assessed by obtaining the BMI using the body height and body mass data. The following formula was used:

BMI = (body mass [kg])/(body height [m])^2^ (1)

The results were classified as shown in Table 1.

### 2.3. Inclusion and Exclusion Criteria

To participate in the research, participants (18 years old or older) were required to complete the whole questionnaire. Participation was voluntary and equivalent to agreeing to the use of the obtained data for research purposes (which was disclosed to the participants).

### 2.4. Statistical Analysis

The results underwent statistical analysis to establish whether the change in the frequency of meat consumption was influenced by age, sex, nutritional status, residency, level of education, occupation, and form of work during the lockdown period.

The only continuous variable was age. To examine the compliance of age with normal distribution, the Shapiro–Wilk test and visual histogram analysis were used. Because the age distribution did not follow a normal distribution, the median was used as a measure of central tendency, and the interquartile range (IQR) was used as a measure of dispersion.

The χ^2^ test was used to examine the independence of qualitative variables. Variables for which a significant relationship with increasing, or decreasing the consumption of specific categories of food products, was found in the χ^2^ test were further analyzed using univariate and multivariate logistic regression. A *p* value lower than 0.05 was defined as statistically significant.

For statistical analyses, the R software (version 4.0.4, R Core Team (2021). R: A language and environment for statistical computing. R Foundation for Statistical Computing, Vienna, Austria. URL https://www.R-project.org/, accessed on 1 March 2021) and Statistica [“TIBCO Software Inc., Santa Clara, CA, USA (2017). Statistica (data analysis software system), version 13. http://statistica.io, accessed on 18 July 2023”] were used.

### 2.5. Ethical Aspects

The study was conducted in compliance with the applicable legal standards and general ethical and deontological principles. An inquiry was submitted to the Bioethics Committee, and the response was that conducting the study in accordance with the planned methodology did not require permission from the bioethics committee (Bioethics Committee of the Medical University of Silesia, BNW/NWN/0052/KB/42/24, 20 February 2024).

## 3. Results

### 3.1. Study Group Characteristics

The study group characteristics are shown in Table 2. The study involved 3888 people, most of whom were women (84.54%). Most of the study participants had a normal body weight (58.74%). The vast majority were people with a high school (40.82%) or university education (57.33%).

### 3.2. Changes in Meat Consumption during the COVID-19 Pandemic

Changes in the frequency of meat consumption were observed during the lockdown period. The percentage of people whose consumption frequency changed was different for particular kinds of meat. The frequency of poultry consumption increased, while for pork, beef, ham and other meat products as well as fish and seafood, it declined (Table 3).

#### 3.2.1. Age

A significant relationship was found between age and changes in the consumption of all of tested categories of food products (*p* < 0.001 for each meat and fish category). Poultry was the only product whose consumption increased by a larger percentage of respondents than decreased, and only in the two youngest categories (between 18 and 34 years old).

The percentage of people among whom pork consumption decreased ranged from 18.25% among people aged 18–23 to 45.07% among people aged 55–64. The percentage of people declaring an increase in pork consumption ranged from 8.41% among people aged 45–54 to 14.01% among people aged 18–23. Therefore, people aged 18–23, compared to other age groups, were the least likely to declare a decrease in pork consumption, and the most likely to declare an increase.

The largest decreases in consumption were observed in the case of beef. The highest percentage of people who declared a decrease in beef consumption concerned the group aged 55–64 and amounted to 56.34%. In the same age group, the highest percentage of people declared a reduction in the consumption of ham and other meat products (45.08%). The age group in which the most people declared a decrease in fish and seafood consumption was people aged at least 65 years (47.82%), and the largest percentage that declared an increase in the consumption of these products was in the group of people aged 18–23 (13.09%).

A full presentation of the results on the relationship between the consumption of individual types of meat and age is presented in Table 4. It should be noted that the number of persons in the oldest age categories was the lowest, which may have partially affected the results obtained.

#### 3.2.2. Sex

No statistically significant influence of sex was found on the frequency of poultry consumption. However, in the case of other categories of food products, a significant relationship was found between gender and changes in the frequency of consumption.

For all other categories of food products, the percentage of women who declared a reduced consumption was higher than among the men: 24.33% vs. 19.63% (pork), 28.23% vs. 22.3% (beef), 25.83% vs. 18.3% (ham and other meet products), and 27.2% vs. 18.64% (fish and seafood).

The differences between the percentage of people who declared an increase in the consumption of particular types of products were clearly smaller. In the case of fish and seafood, the value of this percentage was very similar for both sexes (11.08% among women and 10.98% among men). For other product categories, however, the percentage of people declaring an increase in consumption was clearly higher among men than among women: 13.31% vs. 10.65% (pork), 11.51% vs. 7.24% (beef) and 15.64% vs. 13.35% (ham and other meat products).

A full presentation of the results on the relationship between the consumption of individual types of meat and sex is presented in Table 5.

#### 3.2.3. BMI Category according to the WHO

Patients with a higher BMI value declared a less frequent consumption of pork, beef, ham and other meat products as well as fish and seafood. Regarding poultry, the same tendency was observed except for the underweight group, in which the same percentage of people increased and decreased their poultry consumption. A full presentation of the results on the relationship between the consumption of different types of meat and fish during the COVID-19 lockdown period and BMI category is presented in Table 6.

#### 3.2.4. Place of Residence

In the group of residents from the biggest cities, a lower percentage of people decreased their beef consumption than in other areas. Place of residence did not influence the frequency of consumption of other categories of products. A full presentation of the results on the relationship between the consumption of types of meat and fish during the COVID-19 lockdown period and place of residence is presented in Table 7.

#### 3.2.5. Level of Education

No statistically significant differences were found in the groups with different levels of education (Table 8).

#### 3.2.6. Occupation

Retired people and pensioners were the group most likely to limit their consumption of meat of every category. Students were the most likely out of all groups to increase their consumption of meat of every category. A full presentation of the results on the relationship between the consumption of individual types of meat and fish during the COVID-19 lockdown period and occupation is presented in Table 9.

#### 3.2.7. Form of Work during Lockdown Period

A significant relationship was found between the consumption of meat and fish and the form of work during the lockdown period for all meat and fish categories included.

The highest percentage of people whose chicken consumption increased was recorded among people who were not working (20.26%), while the lowest percentage of people whose chicken consumption increased was among people from the partially remote, partially non-remote work group (11.11%). The percentage of people who stated a decrease in chicken consumption was less diverse depending on the form of work during the pandemic and ranged from 16.85% among people from the remote work group to 18.52% among people from the partially remote, partially non-remote work group.

In terms of other categories of meat and fish (pork, beef, ham and other meat products, fish and seafood), in each group of people in terms of the form of work during the pandemic, the percentage of people who stated a decrease in the consumption of a given product category was higher than the percentage of people who stated an increase in the consumption of a given product category.

A full presentation of the results on the relationship between the consumption of individual types of meat and fish and form of work during lockdown period is presented in Table 10.

### 3.3. Logistic Regression

#### 3.3.1. Factors Influencing the Increase in Consumption of Particular Product Categories

A multivariate logistic regression model showed that the increase in the consumption of poultry, pork, beef as well as fish and seafood was influenced not by a younger age, but by the status of a student. The exception was the increased frequency of the consumption of ham and other meat products, which the multivariate model correlated with both the status of a student and younger age. Working partly remotely and partly at the workplace in the logistic regression models turned out to have no statistically significant impact on the increase in the frequency of fish and seafood consumption.

A complete summary of the results of the logistic regression analysis regarding the examination of factors that could increase the risk of the increased consumption of specific products is presented in Table 11.

#### 3.3.2. Factors Influencing the Decrease in Consumption of Particular Product Categories

The analysis of the multivariate models showed that the following factors contributed to the decline in the consumption of all categories of meat products: older age, female sex, and BMI status indicating overweight or obesity. In the case of all categories except beef, the decline in product consumption was not influenced by the pensioner’s status, but rather by older age itself. Living outside the largest cities with more than 250,000 inhabitants was actually predisposed to a reduction in beef consumption.

A complete summary of the results of the logistic regression analysis regarding the examination of factors that could decrease the risk of increased consumption of specific products is presented in Table 12.

## 4. Discussion

Papers describing the influence of the COVID-19 pandemic on the dietary behaviors of Polish people have also addressed the frequency of meat consumption. Sidor and Rzymski conducted a study with 1097 participants that showed that the factors differentiating the frequency of eating meat during the pandemic were sex, age, and BMI category. According to their study, men consumed meat more often than women [29]. Their finding is consistent with the results of this research and with the general trend applicable to populations of many countries: in general, women tend to eat less meat than men. A similar tendency has also been observed in China [30]. The differences in the amount of meat eaten by men and women is connected to personality characteristics and individual values [31,32,33].

According to the research by Sidor and Rzymski, people above the age of 45 years are the group most likely out of all age groups to increase their meat consumption frequency. Furthermore, it has been shown that people with overweight and obesity increased their meat consumption significantly more than people with a lower BMI [29]. A higher consumption of meat in people with overweight and obesity in Poland was also noted in the analysis by Leszczak et al. [34] The reason for this inconsistency with our research remains unclear. It is possible that the discrepancy in the results is a result of the different characteristics of the study groups. Due to this, it seems that in order to examine the dietary behaviors of people with overweight and obesity more thoroughly, further research is necessary. Sidor and Rzymski emphasize the need to support people with excess body weight [29]. Because of the risk of weight gain during the pandemic, such people are more susceptible to a higher severity of coronavirus infection.

Błaszczyk-Bębenek et al. [35] mentioned that in the group of Polish people that they examined in the lockdown period, the consumption of canned meat became more frequent, whereas the frequency of red meat consumption decreased. The data concerning red meat are compatible with the results of this study; however, it contradicts our results regarding ham and other meat products, which according to this study, were consumed less frequently during the lockdown period in general. A possible cause for such discrepancy may be classifying canned meat in one category with other products, while in the research of Błaszczyk-Bębenek et al., the canned meat was treated as a distinct category [35].

In general, both Sidor and Rzymski [29] as well as Błaszczyk-Bębenek et al. [35] suggest that the COVID-19 lockdown period did not permanently change the dietary behaviors of Polish people.

There is a lack of detailed research about the factors differentiating the amount of meat consumed in Poland during the COVID-19 pandemic. However, after a thorough analysis of our results, it can be beneficial to outline possible reasons for the changes in the levels of meat consumption. Numerous papers concerning dietary patterns during the COVID-19 pandemic in countries other than Poland point to the fact that a decrease in meat consumption might have been a result of a lack of accessibility due to problems in the meat product market and higher prices.

In the article describing the influence of COVID-19 regulations on dietary behaviors in Spain, Rodriguez-Perez et al. mentioned that the frequency of meat consumption in the Spanish population became lower. A possible explanation for this decline in meat consumption in 28% of the study participants was the lack of accessible meat in Spanish stores [36].

Another community in which a decrease in meat consumption was observed was in Indian society. Faslu Rahman et al. claimed that 33% of participants who used to eat meat before the pandemic later changed their diet to vegetarian during the lockdown period. This was the result of the disrupted production and transport of meat products, which led to more difficulty in terms of access and an increase in the prices of meat products [37]. At the beginning of the COVID-19 pandemic, although the general opinion about meat consumption had been negative because it seemed to be connected to an increased risk of SARS-CoV-2 infection, after a while, dietary patterns returned to their previous state. However, because of the decrease in the availability of meat, only 46.4% of the study participants were able to buy enough meat to fulfill their needs. Other people from this study group where unable to buy meat, or the amount of meat that they bought was insufficient. The most available kind of meat was poultry, which could have been bought by 81% of the study group, while the least available kind of meat was pork (only 7% of participants were able to buy it during lockdown). The production of meat in India before the pandemic was mostly local, and therefore during the lockdowns, the availability and level of consumption varied in different regions [37].

Haskacara et al. conducted a study to examine the influence of the COVID-19 pandemic on the consumption of meat in Turkey. A total of 13% of participants declared lowering their level of red meat consumption, 11% declared decreased poultry consumption, and 31% declared decreased fish consumption. The reason behind the decreased red meat and poultry consumption seems to be economic, while the decline in fish consumption was due to poor availability [38]. Kartari and et al. also pointed to the decrease in fish and meat consumption in Turkey during that period [39].

Kartari et al. also examined the populations of Portugal and China. A higher level of fish consumption was noted in Portugal, where fish and seafood are commonly eaten every day and are a part of Portuguese culture [39]. A higher meat consumption was observed in the Chinese population [39].

According to Husain and Ashkanani, the decrease in the frequency of fish and seafood consumption in Kuwait was caused by difficulties in the production and market [40], similarly to the situation in China [41]. Revoredo-Giha et al. described an increased demand for meat in the United Kingdom, where in the first days of lockdown, British people spent 16% more on meat and fish, which brought the level of sales to a state similar to the previous Christmas period. Nevertheless, larger purchases are not equivalent to the rise in consumption, and a possible explanation could be a desire to accumulate food out of fear of the future [42,43].

Given the above examples, we can assume that the differentiation of meat consumption in Poland in groups of different occupations and places of residence may be due to the different availability of meat products. Residents in the largest cities generally have access to a large number of stores and restaurants, which could explain the lower rates of decline in beef consumption during lockdown in comparison to the other groups.

Retirement or being a pensioner seemed to be a factor that increased the probability of lowering the consumption of every kind of meat. It is worth noting that since the beginning of the COVID-19 pandemic, mortality due to COVID-19 was significantly higher in elderly people [44]. Such information was made available to the public, and elderly people were advised to limit their time spent in public places to a minimum, in order to protect them from severe coronavirus infections as one of the most vulnerable groups. We can assume that a decrease in meat consumption in the group of retired people and pensioners was a result of adverse circumstances preventing them from sufficient grocery shopping.

There is a lack of studies analyzing the influence of the form of work undertaken during the lockdown period on meat consumption. In this study, the group who worked partly remotely and partly not remotely seemed to show different trends than the other groups, but the logistic regression model did not confirm a significant relationship between this form of work and changes in the consumption of particular types of meat. It was, however, a much smaller group than those who performed just remote work, just non-remote work, or were unemployed. Furthermore, in other groups, no tendency regarding meat consumption was observed, and therefore the conclusions are unclear.

Since the beginning, the COVID-19 pandemic influenced the mental health of people in a negative way [45]. The correlation between emotional state and food choices was reflected by the increase in the consumption of carbohydrates and fats as well as the increased demand for “fast-food” such as hamburgers during the pandemic [18,46].

However, societies in which a healthy lifestyle and well-balanced diet are included were firmly grounded before the pandemic, and so tended to make better food choices during the pandemic. A great example is Italy, where the popularity of the Mediterranean diet did not decrease during the lockdown period [47,48,49]. Ruotolo et al. stated that in comparison to 2019, in 2020 in Italy, there was a decrease in the consumption of red meat (−15%), pork and canned meat (−29%), poultry (−26%), and fish (−25%). One of the reasons for such food choices was the raised awareness and preference toward products of best quality and nutritional value such as vegetables, fruit, and pasta [50]. Similarly, in Portugal and Turkey, the tendency to maintain a Mediterranean diet in everyday life was observed during the COVID-19 pandemic [39].

Nevertheless, even in Italy, where the popularity of vegetables, legumes, and fruit is increased, among adolescents, the consumption of highly processed food and so called “comfort foods” (e.g., chocolate, sweet packed snacks, ice cream, desserts, or bread) increased. This was linked to weight gain in adolescents, whose growth in height had finished. Children and adolescents were described as particularly vulnerable to stress in the lockdown period which resulted in mental health problems and the increased consumption of “comfort foods” [51]. The feeling of boredom and emptiness led to similar dietary changes in the population of Saudi Arabia, despite the increase in the consumption of fruit and vegetables [52].

### Strengths and Limitations of the Study

We believe that the publication prepared by our team has some important strengths. The strength of the study presented in this publication is that the analysis of changes in the consumption of meat and fish was divided into various categories of these products depending on a number of factors such as age, gender, education, BMI category, place of residence, type, and form of work performed. Moreover, the group of respondents was quite large, and the data were collected within a short enough period of time so that the epidemic restrictions did not change significantly.

Our study also had some limitations. The period of restrictions related to the COVID-19 pandemic had various phases. Our study only looked at the beginning of the pandemic. Therefore, it is not possible to know how people’s dietary habits changed over the further course of the pandemic in the same group of respondents. Another limitation was the methodology of collecting the information. The nature of the study referred only to the relationship between changes in meat or fish consumption and selected variables relating to the characteristics of the studied population at a given time. No conclusions about cause and effect can be drawn from this study. Moreover, the collected data were largely based on the subjective assessment of the respondents as the study was conducted in the form of a survey via social media. It cannot be ruled out that some respondents answered the questionnaire in a less thoughtful way than if the survey had been conducted with the participation of an interviewer. On the other hand, this form of conducting a survey probably allowed us to reached a larger group of respondents.

## 5. Conclusions

The results of this research show that the factors that differentiated the frequency of meat consumption during the lockdown period were age, sex, occupation, place of residence, and BMI category. The level of education and form of work during the pandemic did not influence the frequency of meat product consumption. The factors shaping the dietary behaviors of Polish people are complex, and further research is needed to understand the patterns of meat consumption.

Based on our observations and the conclusions of other researchers, we can assume that the changes in meat consumption in Poland were a result of the accessibility of different meats for various groups of people. However, a raised awareness regarding a healthy lifestyle would be beneficial to society, especially for people with excess body weight. To follow a well-balanced diet in stressful circumstances such as the COVID-19 pandemic, individuals need to be previously educated about it and perceive a healthy diet as having value.

Last but not least, special care needs to be provided to elderly people to whom food may be inaccessible due to geographical reasons, which may be a risk to their food safety.

## Figures and Tables

**Table 1:** Classification of nutritional status (BMI according to the WHO).

BMI [kg/m^2^]	Status
<18.5	Underweight
18.5–24.9	Normal body mass
25.0–29.9	Overweight
≥30.0	Obese

**Table 2:** Study group characteristics.

Parameter		Value
Total amount of participants	N	3888
Age *	Median	27
	IQR	23–35
Sex	Female	3287 (84.54%)
	Male	601 (15.46%)
BMI category according to the WHO	Underweight	178 (4.58%)
	Normal weight	2284 (58.74%)
	Overweight	967 (24.87%)
	Obesity	459 (11.81%)
Current place of residence	Rural area	872 (22.43%)
	Urban area up to 50,000 population	648 (16.67%)
	Urban area 50–100,000 population	482 (12.40%)
	Urban area 100–250,000 population	611 (15.72%)
	Urban area over 250,000 population	1275 (32.79%)
Level of education	Primary school	28 (0.72%)
	Middle school	44 (1.13%)
	High school	1587 (40.82%)
	University degree	2229 (57.33%)
Occupation	Blue collar worker	781 (20.09%)
	White collar worker	1813 (46.63%)
	Unemployed	281 (7.23%)
	Retired/Pensioner	56 (1.44%)
	Student	957 (24.61%)
Form of work during lockdown period	Did not work	1816 (46.71%)
	Non-remote work	926 (23.82%)
	Remote work	1092 (28.09%)
	Partially remote, partially non-remote work	54 (1.39%)

* Shapiro–Wilk normality test, *p* < 0.001.

**Table 3:** The frequency of consumption of different kinds of meat during the COVID-19 lockdown period compared to the time period before the pandemic.

Frequency of Consumption	Category of Product				
	Poultry (N = 3888)	Pork (N = 3888)	Beef (N = 3888)	Ham and Other Meat Products (N = 3888)	Fish and Seafood (N = 3888)
Much less frequently	248 (6.38%)	431 (11.09%)	586 (15.07%)	468 (12.04%)	523 (13.45%)
Slightly less frequently	413 (10.62%)	487 (12.52%)	476 (12.24%)	497 (12.78%)	483 (12.42%)
The same as before	2521 (64.84%)	2540 (65.33%)	2520 (64.81%)	2390 (61.47%)	2452 (63.07%)
Slightly more frequently	494 (12.71%)	332 (8.54%)	246 (6.34%)	385 (9.90%)	323 (8.31%)
Much more frequently	212 (5.45%)	98 (2.52%)	60 (1.54%)	148 (3.81%)	107 (2.75%)

**Table 4:** Changes in the frequency of meat consumption during the COVID-19 lockdown period compared to the time before the pandemic in different age groups. * means a significant dependence between changes in consumption of a given type of meat or fish and age (χ^2^ test).

Category of Product		Age [years]						*p*
		18–23 (N = 1085)	24–34 (N = 1756)	35–44 (N = 739)	45–54 (N = 214)	55–64 (N = 71)	≥65 (N = 23)	
Poultry	Much less frequently	55 (5.07%)	113 (6.44%)	48 (6.50%)	23 (10.75%)	6 (8.45%)	3 (13.04%)	<0.001 *
	Slightly less frequently	103 (9.49%)	161 (9.17%)	90 (12.18%)	38 (17.76%)	15 (21.13%)	6 (26.09%)	
	The same as before	691 (63.69%)	1171 (66.69%)	490 (66.31%)	120 (56.07%)	38 (53.52%)	11 (47.83%)	
	Slightly more frequently	172 (15.85%)	220 (12.53%)	71 (9.61%)	24 (11.21%)	6 (8.45%)	1 (4.35%)	
	Much more frequently	64 (5.90%)	91 (5.18%)	40 (5.41%)	9 (4.21%)	6 (8.45%)	2 (8.70%)	
Pork	Much less frequently	89 (8.20%)	184 (10.48%)	108 (14.61%)	34 (15.89%)	14 (19.72%)	2 (8.70%)	<0.001 *
	Slightly less frequently	109 (10.05%)	211 (12.02%)	105 (14.21%)	38 (17.76%)	18 (25.35%)	6 (26.90%)	
	The same as before	735 (67.74%)	1183 (67.37%)	453 (61.30%)	124 (57.94%)	33 (46.48%)	12 (52.17%)	
	Slightly more frequently	121 (11.15%)	144 (8.20%)	50 (6.77%)	12 (5.61%)	3 (4.23%)	2 (8.70%)	
	Much more frequently	31 (2.86%)	34 (1.94%)	23 (3.11%)	6 (2.80%)	3 (4.23%)	1 (4.35%)	
Beef	Much less frequently	118 (10.88%)	240 (13.67%)	151 (20.43%)	53 (24.77%)	21 (29.58%)	3 (13.04%)	<0.001 *
	Slightly less frequently	125 (11.52%)	202 (11.50%)	92 (12.45%)	31 (14.49%)	19 (26.76%)	7 (30.43%)	
	The same as before	737 (67.93%)	1180 (67.20%)	446 (60.35%)	120 (56.07%)	27 (38.03%)	10 (43.48%)	
	Slightly more frequently	86 (7.93%)	115 (6.55%)	34 (4.60%)	6 (2.80%)	3 (4.23%)	2 (8.70%)	
	Much more frequently	19 (1.75%)	19 (1.08%)	16 (2.17%)	4 (1.87%)	1 (1.41%)	1 (4.35%)	
Ham and other meat products	Much less frequently	100 (9.22%)	185 (10.54%)	116 (15.70%)	45 (21.03%)	16 (22.54%)	6 (26.09%)	<0.001 *
	Slightly less frequently	110 (10.14%)	212 (12.07%)	117 (15.83%)	39 (18.22%)	16 (22.54%)	3 (13.04%)	
	The same as before	684 (63.04%)	1124 (64.01%)	428 (57.92%)	111 (51.87%)	32 (45.07%)	11 (47.83%)	
	Slightly more frequently	146 (13.46%)	171 (9.74%)	52 (7.04%)	9 (4.21%)	5 (7.04%)	2 (8.70%)	
	Much more frequently	45 (4.15%)	64 (3.64%)	26 (3.52%)	10 (4.67%)	2 (2.82%)	1 (4.35%)	
Fish and seafood	Much less frequently	122 (11.24%)	230 (13.10%)	117 (15.83%)	36 (16.82%)	14 (19.72%)	4 (17.39%)	<0.001 *
	Slightly less frequently	112 (10.32%)	211 (12.02%)	95 (12.86%)	43 (20.09%)	15 (21.13%)	7 (30.43%)	
	The same as before	709 (65.35%)	1130 (64.35%)	448 (60.62%)	119 (55.61%)	36 (50.70%)	10 (43.48%)	
	Slightly more frequently	114 (10.51%)	140 (7.97%)	55 (7.44%)	12 (5.61%)	1 (1.41%)	1 (4.35%)	
	Much more frequently	28 (2.58%)	45 (2.56%)	24 (3.25%)	4 (1.87%)	5 (7.04%)	1 (4.35%)	

**Table 5:** Differences in frequency of meat consumption during the COVID-19 lockdown period compared to the time before the pandemic in women and men. * means a significant dependence between changes in consumption of a given type of meat or fish and sex (Chi square test).

Category of Product		Sex		*p*
		Women (N = 3287)	Men (N = 601)	
Poultry	Much less frequently	219 (6.66%)	29 (4.83%)	0.496
	Slightly less frequently	349 (10.62%)	64 (10.65%)	
	The same as before	2123 (64.59%)	398 (66.22%)	
	Slightly more frequently	414 (12.60%)	80 (13.31%)	
	Much more frequently	182 (5.54%)	30 (4.99%)	
Pork	Much less frequently	388 (11.80%)	43 (7.15%)	0.002 *
	Slightly less frequently	412 (12.53%)	75 (12.48%)	
	The same as before	2137 (65.01%)	403 (67.05%)	
	Slightly more frequently	264 (8.03%)	68 (11.31%)	
	Much more frequently	86 (2.62%)	12 (2.00%)	
Beef	Much less frequently	530 (16.12%)	56 (9.32%)	<0.001 *
	Slightly less frequently	398 (12.11%)	78 (12.98%)	
	The same as before	2121 (64.53%)	399 (66.39%)	
	Slightly more frequently	189 (5.75%)	57 (9.48%)	
	Much more frequently	49 (1.49%)	11 (1.83%)	
Ham and other meat products	Much less frequently	420 (12.78%)	48 (7.99%)	0.004 *
	Slightly less frequently	429 (13.05%)	68 (11.31%)	
	The same as before	1999 (60.82%)	391 (65.06%)	
	Slightly more frequently	313 (9.52%)	72 (11.98%)	
	Much more frequently	126 (3.83%)	22 (3.66%)	
Fish and seafood	Much less frequently	470 (14.30%)	53 (8.82%)	<0.001 *
	Slightly less frequently	424 (12.90%)	59 (9.82%)	
	The same as before	2029 (61.73%)	423 (70.38%)	
	Slightly more frequently	273 (8.31%)	50 (8.32%)	
	Much more frequently	91 (2.77%)	16 (2.66%)	

**Table 6:** Differences in frequency of meat consumption during the COVID-19 lockdown period compared to the time before the pandemic in people of different BMI category according to the WHO. * means a significant dependence between changes in consumption of a given type of meat or fish and BMI category (χ^2^ test).

Category of Product		BMI Category According to the WHO				*p*
		Underweight (N = 178)	Normal Weight (N = 2284)	Overweight (N = 967)	Obesity (N = 459)	
Poultry	Much less frequently	13 (7.30%)	140 (6.13%)	65 (6.72%)	30 (6.54%)	0.005 *
	Slightly less frequently	24 (13.48%)	214 (9.37%)	109 (11.27%)	66 (14.38%)	
	The same as before	103 (57.87%)	1527 (66.86%)	623 (64.43%)	268 (58.39%)	
	Slightly more frequently	31 (17.42%)	291 (12.74%)	112 (11.58%)	60 (13.07%)	
	Much more frequently	7 (3.93%)	112 (4.90%)	58 (6.00%)	35 (7.63%)	
Pork	Much less frequently	21 (11.80%)	229 (10.03%)	126 (13.03%)	55 (11.98%)	<0.001 *
	Slightly less frequently	17 (9.55%)	255 (11.16%)	131 (13.55%)	84 (18.30%)	
	The same as before	121 (67.98%)	1554 (68.04%)	600 (62.05%)	265 (57.73%)	
	Slightly more frequently	17 (9.55%)	196 (8.58%)	84 (8.69%)	35 (7.63%)	
	Much more frequently	2 (1.12%)	50 (2.19%)	26 (2.69%)	20 (4.36%)	
Beef	Much less frequently	24 (13.48%)	307 (13.44%)	152 (15.72%)	103 (22.44%)	<0.001 *
	Slightly less frequently	18 (10.11%)	249 (10.90%)	133 (13.75%)	76 (16.56%)	
	The same as before	119 (66.85%)	1537 (67.29%)	610 (63.08%)	254 (55.34%)	
	Slightly more frequently	16 (8.99%)	157 (6.87%)	55 (5.69%)	18 (3.92%)	
	Much more frequently	1 (0.56%)	34 (1.49%)	17 (1.76%)	8 (1.74%)	
Ham and other meat products	Much less frequently	17 (9.55%)	249 (10.90%)	138 (14.27%)	64 (13.94%)	<0.001 *
	Slightly less frequently	20 (11.24%)	273 (11.95%)	125 (12.93%)	79 (17.21%)	
	The same as before	116 (65.17%)	1463 (64.05%)	557 (57.60%)	254 (55.34%)	
	Slightly more frequently	21 (11.80%)	227 (9.94%)	99 (10.24%)	38 (8.28%)	
	Much more frequently	4 (2.25%)	72 (3.15%)	48 (4.96%)	24 (5.23%)	
Fish and seafood	Much less frequently	17 (9.55%)	283 (12.39%)	137 (14.17%)	86 (18.74%)	0.002 *
	Slightly less frequently	23 (12.92%)	265 (11.60%)	123 (12.72%)	72 (15.69%)	
	The same as before	111 (62.36%)	1478 (64.71%)	606 (62.67%)	257 (55.99%)	
	Slightly more frequently	21 (11.80%)	201 (8.80%)	70 (7.24%)	31 (6.75%)	
	Much more frequently	6 (3.37%)	57 (2.50%)	31 (3.21%)	13 (2.83%)	

**Table 7:** Differences in frequency of meat consumption during the COVID-19 lockdown period compared to the time before the pandemic in people of different place of residence. * means a significant dependence between changes in consumption of a given type of meat or fish and current place of residence (χ^2^ test).

Category of Product		Current Place of Residence					*p*
		Rural Area (N = 872)	Urban Area, <50 k (N = 648)	Urban Area, 50–100 k (N = 482)	Urban Area, 100–250 k (N = 611)	Urban Area, >250 k (N = 1275)	
Poultry	Much less frequently	47 (5.39%)	40 (6.17%)	34 (7.05%)	39 (6.38%)	88 (6.90%)	0.863
	Slightly less frequently	96 (11.01%)	66 (10.19%)	49 (10.17%)	76 (12.44%)	126 (9.88%)	
	The same as before	563 (64.56%)	435 (67.13%)	304 (63.07%)	393 (64.32%)	826 (64.78%)	
	Slightly more frequently	114 (13.07%)	76 (11.73%)	63 (13.07%)	76 (12.44%)	165 (12.94%)	
	Much more frequently	52 (5.96%)	31 (4.78%)	32 (6.64%)	27 (4.42%)	70 (5.49%)	
Pork	Much less frequently	86 (9.86%)	77 (11.88%)	64 (13.28%)	74 (12.11%)	130 (10.20%)	0.444
	Slightly less frequently	100 (11.47%)	82 (12.65%)	65 (13.49%)	89 (14.57%)	151 (11.84%)	
	The same as before	584 (66.97%)	416 (64.20%)	302 (62.66%)	389 (63.67%)	849 (66.59%)	
	Slightly more frequently	77 (8.83%)	52 (8.02%)	42 (8.71%)	50 (8.18%)	111 (8.71%)	
	Much more frequently	25 (2.87%)	21 (3.24%)	9 (1.87%)	9 (1.47%)	34 (2.67%)	
Beef	Much less frequently	134 (15.37%)	104 (16.05%)	88 (18.26%)	98 (16.04%)	162 (12.71%)	0.029 *
	Slightly less frequently	100 (11.47%)	82 (12.65%)	61 (12.66%)	90 (14.73%)	143 (11.22%)	
	The same as before	568 (65.14%)	425 (65.59%)	288 (59.75%)	378 (61.87%)	861 (67.53%)	
	Slightly more frequently	53 (6.08%)	27 (4.17%)	39 (8.09%)	39 (6.38%)	88 (6.90%)	
	Much more frequently	17 (1.95%)	10 (1.54%)	6 (1.24%)	6 (0.98%)	21 (1.65%)	
Ham and other meat products	Much less frequently	100 (11.47%)	77 (11.88%)	64 (13.28%)	81 (13.26%)	146 (11.45%)	0.068
	Slightly less frequently	101 (11.58%)	91 (14.04%)	76 (15.77%)	74 (12.11%)	155 (12.16%)	
	The same as before	540 (61.93%)	401 (61.88%)	280 (58.09%)	383 (62.68%)	786 (61.65%)	
	Slightly more frequently	91 (10.44%)	50 (7.72%)	39 (8.09%)	60 (9.82%)	145 (11.37%)	
	Much more frequently	40 (4.59%)	29 (4.48%)	23 (4.77%)	13 (2.13%)	43 (3.37%)	
Fish and seafood	Much less frequently	121 (13.88%)	82 (12.65%)	84 (17.43%)	82 (13.42%)	154 (12.08%)	0.061
	Slightly less frequently	112 (12.84%)	88 (13.58%)	59 (12.24%)	91 (14.89%)	133 (10.43%)	
	The same as before	544 (62.39%)	420 (64.81%)	286 (59.34%)	374 (61.21%)	828 (64.94%)	
	Slightly more frequently	73 (8.37%)	40 (6.17%)	41 (8.51%)	46 (7.53%)	123 (9.65%)	
	Much more frequently	22 (2.52%)	18 (2.78%)	12 (2.49%)	18 (2.95%)	37 (2.90%)	

**Table 8:** Frequency of meat consumption during the COVID-19 lockdown period compared to the time before the pandemic in people of different levels of education.

Category of Product		Level of Education			*p*
		Primary and Middle School (N = 72)	High School (N = 1587)	University Degree (N = 2229)	
Poultry	Much less frequently	7 (9.72%)	98 (6.18%)	143 (6.42%)	0.099
	Slightly less frequently	13 (18.06%)	165 (10.40%)	235 (10.54%)	
	The same as before	38 (52.78%)	1008 (63.52%)	1475 (66.17%)	
	Slightly more frequently	10 (13.89%)	216 (13.61%)	268 (12.02%)	
	Much more frequently	4 (5.56%)	100 (6.30%)	108 (4.85%)	
Pork	Much less frequently	11 (15.28%)	167 (10.52%)	253 (11.35%)	0.462
	Slightly less frequently	10 (13.89%)	201 (12.67%)	276 (12.38%)	
	The same as before	47 (65.28%)	1023 (64.46%)	1470 (65.95%)	
	Slightly more frequently	3 (4.17%)	152 (9.58%)	177 (7.94%)	
	Much more frequently	1 (1.39%)	44 (2.77%)	53 (2.38%)	
Beef	Much less frequently	13 (18.06%)	240 (15.12%)	333 (14.94%)	0.527
	Slightly less frequently	11 (15.28%)	208 (13.11%)	257 (11.53%)	
	The same as before	44 (61.11%)	1002 (63.14%)	1474 (66.13%)	
	Slightly more frequently	3 (4.17%)	108 (6.81%)	135 (6.06%)	
	Much more frequently	1 (1.39%)	29 (1.83%)	30 (1.35%)	
Ham and other meat products	Much less frequently	8 (11.11%)	188 (11.85%)	272 (12.20%)	0.684
	Slightly less frequently	9 (12.50%)	213 (13.42%)	275 (12.34%)	
	The same as before	45 (62.50%)	949 (59.80%)	1396 (62.63%)	
	Slightly more frequently	8 (11.11%)	169 (10.65%)	208 (9.33%)	
	Much more frequently	2 (2.78%)	68 (4.28%)	78 (3.50%)	
Fish and seafood	Much less frequently	16 (22.22%)	219 (13.80%)	288 (12.92%)	0.089
	Slightly less frequently	10 (13.89%)	181 (11.41%)	292 (13.10%)	
	The same as before	44 (61.11%)	1002 (63.14%)	1406 (63.08%)	
	Slightly more frequently	0 (0.00%)	138 (8.70%)	185 (8.30%)	
	Much more frequently	2 (2.78%)	47 (2.96%)	58 (2.60%)	

**Table 9:** Frequency of meat consumption during the COVID-19 lockdown period compared to the time before the pandemic in people of different occupations. * means a significant dependence between changes in consumption of a given type of meat or fish and occupation (χ^2^ test).

Category of Product		Occupation					*p*
		Blue Collar Worker (N = 781)	White Collar Worker (N = 1813)	Unemployed (N = 281)	Retired/Pensioner (N = 56)	Student (N = 957)	
Poultry	Much less frequently	51 (6.53%)	123 (6.78%)	14 (4.98%)	5 (8.93%)	55 (5.75%)	<0.001 *
	Slightly less frequently	96 (12.29%)	178 (9.82%)	31 (11.03%)	16 (28.57%)	92 (9.61%)	
	The same as before	506 (64.79%)	1200 (66.19%)	191 (67.97%)	28 (50.00%)	596 (62.28%)	
	Slightly more frequently	84 (10.76%)	219 (12.08%)	29 (10.32%)	3 (5.36%)	159 (16.61%)	
	Much more frequently	44 (5.63%)	93 (5.13%)	16 (5.69%)	4 (7.14%)	55 (5.75%)	
Pork	Much less frequently	94 (12.04%)	214 (11.80%)	31 (11.03%)	10 (17.86%)	82 (8.57%)	<0.001 *
	Slightly less frequently	101 (12.93%)	227 (12.52%)	38 (13.52%)	17 (30.36%)	104 (10.87%)	
	The same as before	505 (64.66%)	1186 (65.42%)	191 (67.97%)	24 (42.86%)	634 (66.25%)	
	Slightly more frequently	59 (7.55%)	142 (7.83%)	16 (5.69%)	3 (5.36%)	112 (11.70%)	
	Much more frequently	22 (2.82%)	44 (2.43%)	5 (1.78%)	2 (3.57%)	25 (2.61%)	
Beef	Much less frequently	139 (17.80%)	270 (14.89%)	51 (18.15%)	18 (32.14%)	108 (11.29%)	<0.001 *
	Slightly less frequently	99 (12.68%)	215 (11.86%)	31 (11.03%)	17 (30.36%)	114 (11.91%)	
	The same as before	479 (61.33%)	1203 (66.35%)	186 (66.19%)	18 (32.14%)	634 (66.25%)	
	Slightly more frequently	51 (6.53%)	99 (5.46%)	8 (2.85%)	3 (5.36%)	85 (8.88%)	
	Much more frequently	13 (1.66%)	26 (1.43%)	5 (1.78%)	0 (0.00%)	16 (1.67%)	
Ham and other meat products	Much less frequently	94 (12.04%)	230 (12.69%)	28 (9.96%)	17 (30.36%)	99 (10.34%)	<0.001 *
	Slightly less frequently	112 (14.34%)	228 (12.58%)	39 (13.88%)	11 (19.64%)	107 (11.18%)	
	The same as before	491 (62.87%)	1111 (61.28%)	188 (66.90%)	21 (37.50%)	579 (60.50%)	
	Slightly more frequently	58 (7.43%)	178 (9.82%)	14 (4.98%)	5 (8.93%)	130 (13.58%)	
	Much more frequently	26 (3.33%)	66 (3.64%)	12 (4.27%)	2 (3.57%)	42 (4.39%)	
Fish and seafood	Much less frequently	119 (15.24%)	246 (13.57%)	51 (18.15%)	12 (21.43%)	95 (9.93%)	<0.001 *
	Slightly less frequently	95 (12.16%)	233 (12.85%)	37 (13.17%)	15 (26.79%)	103 (10.76%)	
	The same as before	489 (62.61%)	1139 (62.82%)	177 (62.99%)	25 (44.64%)	622 (64.99%)	
	Slightly more frequently	54 (6.91%)	147 (8.11%)	10 (3.56%)	2 (3.57%)	110 (11.49%)	
	Much more frequently	24 (3.07%)	48 (2.65%)	6 (2.14%)	2 (3.57%)	27 (2.82%)	

**Table 10:** Frequency of meat consumption during the COVID-19 lockdown period compared to the time before the pandemic in people who worked in different forms at that time. * means a significant dependence between changes in consumption of a given type of meat or fish and form of work during lockdown period (χ^2^ test).

Category of Product		Form of Work during Lockdown Period				*p*
		Did Not Work (N = 1816)	Non-Remote Work (N = 926)	Remote Work (N = 1092)	Partially Remote, Partially Non-Remote Work (N = 54)	
Poultry	Much less frequently	115 (6.33%)	59 (6.37%)	70 (6.41%)	4 (7.41%)	0.026 *
	Slightly less frequently	195 (10.74%)	98 (10.58%)	114 (10.44%)	6 (11.11%)	
	The same as before	1138 (62.67%)	644 (69.55%)	701 (64.19%)	38 (70.37%)	
	Slightly more frequently	257 (14.15%)	84 (9.07%)	148 (13.55%)	5 (9.26%)	
	Much more frequently	111 (6.11%)	41 (4.43%)	59 (5.40%)	1 (1.85%)	
Pork	Much less frequently	198 (10.90%)	112 (12.10%)	110 (10.07%)	11 (20.37%)	<0.001 *
	Slightly less frequently	231 (12.72%)	112 (12.10%)	139 (12.73%)	5 (9.26%)	
	The same as before	1175 (64.70%)	639 (69.01%)	689 (63.10%)	37 (68.52%)	
	Slightly more frequently	162 (8.92%)	50 (5.40%)	119 (10.90%)	1 (1.85%)	
	Much more frequently	50 (2.75%)	13 (1.40%)	35 (3.21%)	0 (0.00%)	
Beef	Much less frequently	286 (15.75%)	140 (15.12%)	150 (13.74%)	10 (18.52%)	0.009 *
	Slightly less frequently	240 (13.22%)	98 (10.58%)	132 (12.09%)	6 (11.11%)	
	The same as before	1141 (62.83%)	640 (69.11%)	703 (64.38%)	36 (66.67%)	
	Slightly more frequently	124 (6.83%)	37 (4.00%)	83 (7.60%)	2 (3.70%)	
	Much more frequently	25 (1.38%)	11 (1.19%)	24 (2.20%)	0 (0.00%)	
Ham and other meat products	Much less frequently	227 (12.50%)	105 (11.34%)	130 (11.90%)	6 (11.11%)	0.003 *
	Slightly less frequently	219 (12.06%)	124 (13.39%)	144 (13.19%)	10 (18.52%)	
	The same as before	1100 (60.57%)	610 (65.87%)	645 (59.07%)	35 (64.81%)	
	Slightly more frequently	191 (10.52%)	62 (6.70%)	130 (11.90%)	2 (3.70%)	
	Much more frequently	79 (4.35%)	25 (2.70%)	43 (3.94%)	1 (1.85%)	
Fish and seafood	Much less frequently	258 (14.21%)	122 (13.17%)	138 (12.64%)	5 (9.26%)	<0.001 *
	Slightly less frequently	239 (13.16%)	96 (10.37%)	144 (13.19%)	4 (7.41%)	
	The same as before	1116 (61.45%)	637 (68.79%)	663 (60.71%)	36 (66.67%)	
	Slightly more frequently	160 (8.81%)	46 (4.97%)	110 (10.07%)	7 (12.96%)	
	Much more frequently	43 (2.37%)	25 (2.70%)	37 (3.39%)	2 (3.70%)	

**Table 11:** The impact of age and having a student status on an increase in the consumption of particular categories of food products. In the case of fish and seafood, partially remote, partially non-remote work is also included.

Category	Variable	Univariate Analysis		Multivariate Analysis	
		OR [95% CI]	*p*	OR [95% CI]	*p*
Poultry	Age	0.98 [0.97–0.99]	0.001	0.99 [0.98–1.002]	0.11
	Student	1.43 [1.19–1.71]	<0.001	1.30 [1.05–1.61]	0.014
Pork	Age	0.20 [0.14–0.28]	0.008	0.99 [0.98–1.007]	0.33
	Student	1.50 [1.21–1.87]	<0.001	1.40 [1.08–1.82]	0.001
Beef	Age	0.98 [0.96–0.99]	0.002	0.98 [0.97–1.003]	0.10
	Student	1.57 [1.22–2.02]	0.0004	1.37 [1.02–1.84]	0.039
Ham and other meat products	Age	0.97 [0.96–0.98]	<0.001	0.98 [0.97–0.99]	0.003
	Student	1.55 [1.28–1.90]	<0.0001	1.28 [1.01–1.61]	0.038
Fish and seafood	Age	0.988 [0.977–0.999]	0.039	0.99 [0.985–1.011]	0.78
	Student	1.50 [1.21–1.87]	<0.001	1.47 [1.14–1.91]	0.003
	Partially remote, partially non-remote work	1.41 [0.66–1.41]	0.38	−−−−−−−−−−−−	

**Table 12:** The impact of different factors on a decrease in the consumption of particular categories of food products.

Category	Variable	Univariate Analysis		Multivariate Analysis	
		OR [95% CI]	*p*	OR [95% CI]	*p*
Poultry	Age	1.26 [1.017–1.034]	<0.0001	1.02 [1.01–1.03]	<0.0001
	Student	2.99 [1.73–5.18]	<0.0001	1.65 [0.90–3.01]	0.11
	Partially remote, partially non-remote work	0.98 [0.56–1.71]	0.93	−−−−−−−−−−−−	
Pork	Age	1.03 [1.023–1.038]	<0.0001	1.02 [1.016–1.034]	<0.0001
	Student	1.32 [1.06–1.63]	0.01	1.38 [1.11–1.72]	0.004
	Overweight/obesity	1.42 [1.22–1.66]	<0.0001	1.30 [1.11–1.53]	0.001
	Retired/pensioner	3.07 [1.81–5.22]	<0.0001	1.49 [0.83–2.66]	0.18
	Partially remote, partially non-remote work	1.24 [0.68–2.28]	0.47	−−−−−−−−−−−−	
Beef	Age	1.03 [1.02–1.04]	<0.0001	1.025 [1.017–1.034]	<0.0001
	Female	1.37 [1.11–1.69]	0.003	1.45 [1.18–1.80]	<0.001
	Overweight/obesity	1.50 [1.30–1.74]	<0.0001	1.36 [1.17–1.59]	<0.0001
	Urban area, >250 k	1.30 [1.11–1.51]	<0.0001	1.26 [1.08–1.48]	0.003
	Retired/pensioner	4.55 [2.63–7.85]	<0.0001	2.16 [1.19–3.90]	0.01
	Partially remote, partially non-remote work	Unable to calculate	0.94	−−−−−−−−−−−−	
Ham and other meat products	Age	1.036 [1.029–1.044]	<0.0001	1.033 [1.024–1.041]	<0.0001
	Female	1.46 [1.17–1.81]	<0.001	1.50 [1.20–1.88]	<0.001
	Overweight/obesity	1.36 [1.17–1.57]	<0.0001	1.20 [1.03–1.41]	0.02
	Retired/pensioner	3.09 [1.82–5.24]	<0.0001	1.26 [0.70–2.26]	0.43
	Partially remote, partially non-remote work	1.28 [0.71–2.30]	0.41	−−−−−−−−−−−−	
Fish and seafood	Age	1.027 [1.019–1.035]	<0.0001	1.022 [1.014–1.031]	<0.0001
	Female	1.63 [1.31–2.03]	<0.0001	1.70 [1.36–2.12]	<0.0001
	Overweight/obesity	1.32 [1.14–1.53]	<0.001	1.41 [0.79–2.51]	0.006
	Retired/pensioner	2.71 [1.60–4.61]	<0.001	1.24 [1.07–1.45]	0.24

## Data Availability

The data presented in this study are available on request from the corresponding author. The data are not publicly available due to privacy.

## References

[1] Lu R., Zhao X., Li J., Niu P., Yang B., Wu H., Wang W., Song H., Huang B., Zhu N. (2020). Genomic characterisation and epidemiology of 2019 novel coronavirus: Implications for virus origins and receptor binding. Lancet.

[2] Lu H., Stratton C.W., Tang Y.W. (2020). Outbreak of pneumonia of unknown etiology in Wuhan, China: The mystery and the miracle. J. Med. Virol..

[3] Jakubiak G.K., Ochab-Jakubiak J., Cieślar G., Stanek A. (2020). Gastrointestinal symptoms in the course of COVID-19. Postepy Hig. Med. Dosw..

[4] Lu S., Huang X., Liu R., Lan Y., Lei Y., Zeng F., Tang X., He H. (2022). Comparison of COVID-19 induced respiratory failure and typical ARDS: Similarities and differences. Front. Med..

[5] Abdel Moneim A., Radwan M.A., Yousef A.I. (2022). COVID-19 and cardiovascular disease: Manifestations, pathophysiology, vaccination, and long-term implication. Curr. Med. Res. Opin..

[6] Hernandez Acosta R.A., Esquer Garrigos Z., Marcelin J.R., Vijayvargiya P. (2022). COVID-19 pathogenesis and clinical manifestations. Infect. Dis. Clin. N. Am..

[7] Lui G., Guaraldi G. (2023). Drug treatment of COVID-19 infection. Curr. Opin. Pulm. Med..

[8] Kaptein F.H.J., Stals M.A.M., Huisman M.V., Klok F.A. (2021). Prophylaxis and treatment of COVID-19 related venous thromboembolism. Postgrad. Med..

[9] Raglow Z., Malani P.N., Petty L.A. (2023). Outpatient treatment for COVID-19. JAMA.

[10] Wang L., Li G., Yuan C., Yang Y., Ling G., Zheng J., Zhou Y., Zhang T., Lin W., Lin Z. (2021). Progress in the diagnosis and treatment of COVID-19 in children: A review. Int. J. Gen. Med..

[11] Sutkowska E., Stanek A., Madziarska K., Jakubiak G.K., Sokołowski J., Madziarski M., Sutkowska-Stępień K., Biernat K., Mazurek J., Borowkow-Bulek A. (2023). Physical activity modifies the severity of COVID-19 in hospitalized patients-observational study. J. Clin. Med..

[12] Rottoli M., Bernante P., Belvedere A., Balsamo F., Garelli S., Giannella M., Cascavilla A., Tedeschi S., Ianniruberto S., Rosselli Del Turco E. (2020). How important is obesity as a risk factor for respiratory failure, intensive care admission and death in hospitalised COVID-19 patients? Results from a single Italian centre. Eur. J. Endocrinol..

[13] Romero Starke K., Reissig D., Petereit-Haack G., Schmauder S., Nienhaus A., Seidler A. (2021). The isolated effect of age on the risk of COVID-19 severe outcomes: A systematic review with meta-analysis. BMJ Glob. Health.

[14] Lim Y., Lee M.H., Jeong S., Han H.W. (2023). Association of physical activity with SARS-CoV-2 infection and severe clinical outcomes among patients in South Korea. JAMA Netw. Open..

[15] Graziani D., Soriano J.B., Del Rio-Bermudez C., Morena D., Díaz T., Castillo M., Alonso M., Ancochea J., Lumbreras S., Izquierdo J.L. (2020). Characteristics and prognosis of COVID-19 in patients with COPD. J. Clin. Med..

[16] Mohamed K., Rzymski P., Islam M.S., Makuku R., Mushtaq A., Khan A., Ivanovska M., Makka S.A., Hashem F., Marquez L. (2022). COVID-19 vaccinations: The unknowns, challenges, and hopes. J. Med. Virol..

[17] Gieseking J., Mangold W., Lewin K. (2014). The people, place, and space Reader. Psychological Ecology (1943).

[18] Ijaz M., Yar M.K., Badar I.H., Ali S., Islam M.S., Jaspal M.H., Hayat Z., Sardar A., Ullah S., Guevara-Ruiz D. (2021). Meat production and supply chain under COVID-19 scenario: Current trends and future prospects. Front. Vet. Sci..

[19] Koronawirus Opinie Polek i Polaków i Ocena Działań Pracodawców. https://odpowiedzialnybiznes.pl/wp-content/uploads/2020/05/FOB_Koronawirus-opinie-Polakow-i-ocena-dzialan-pracodawcow.pdf.

[20] Huang Y., Zhao N. (2020). Generalized anxiety disorder, depressive symptoms and sleep quality during COVID-19 outbreak in China: A web-based cross-sectional survey. Psychiatry Res..

[21] Haddad C., Zakhour M., Bou Kheir M., Haddad R., Al Hachach M., Sacre H., Salameh P. (2020). Association between eating behavior and quarantine/confinement stressors during the coronavirus disease 2019 outbreak. J. Eat. Disord..

[22] Wang C., Pan R., Wan X., Tan Y., Xu L., Ho C.S., Ho R.C. (2020). Immediate psychological responses and associated factors during the initial stage of the 2019 coronavirus disease (COVID-19) epidemic among the general population in China. Int. J. Environ. Res. Public Health.

[23] Sygit-Kowalkowska E. (2014). Coping with stress as a health behavior—Psychological perspectives. Hygeia Public Health.

[24] Battaglia Richi E., Baumer B., Conrad B., Darioli R., Schmid A., Keller U. (2015). Health risks associated with meat consumption: A review of epidemiological studies. Int. J. Vitam. Nutr. Res..

[25] Hamer M., Gale C.R., Kivimäki M., Batty G.D. (2020). Overweight, obesity, and risk of hospitalization for COVID-19: A community-based cohort study of adults in the United Kingdom. Proc. Natl. Acad. Sci. USA.

[26] O’Hearn M., Liu J., Cudhea F., Micha R., Mozaffarian D. (2021). Coronavirus disease 2019 hospitalizations attributable to cardiometabolic conditions in the United States: A comparative risk assessment analysis. J. Am. Heart Assoc..

[27] Elsahoryi N., Al-Sayyed H., Odeh M., McGrattan A., Hammad F. (2020). Effect of Covid-19 on food security: A cross-sectional survey. Clin. Nutr. ESPEN..

[28] Gibb R., Franklinos L.H.V., Redding D.W., Jones K.E. (2020). Ecosystem perspectives are needed to manage zoonotic risks in a changing climate. BMJ.

[29] Sidor A., Rzymski P. (2020). Dietary choices and habits during COVID-19 lockdown: Experience from Poland. Nutrients.

[30] Jia P., Liu L., Xie X., Yuan C., Chen H., Guo B., Zhou J., Yang S. (2021). Changes in dietary patterns among youths in China during COVID-19 epidemic: The COVID-19 impact on lifestyle change survey (COINLICS). Appetite.

[31] Modlinska K., Adamczyk D., Maison D., Pisula W. (2020). Gender differences in attitudes to vegans/vegetarians and their food preferences, and their implications for promoting sustainable dietary patterns—A systematic review. Sustainability.

[32] Mertens A., von Krause M., Meyerhöfer S., Aziz C., Baumann F., Denk A., Heitz T., Maute J. (2020). Valuing humans over animals—Gender differences in meat-eating behavior and the role of the Dark Triad. Appetite.

[33] Hayley A., Zinkiewicz L., Hardiman K. (2015). Values, attitudes, and frequency of meat consumption. Predicting meat-reduced diet in Australians. Appetite.

[34] Leszczak J., Czenczek-Lewandowska E., Wyszyńska J., Weres A., Lewandowski B., Baran J. (2022). Consumption of selected food products by adults representing various body mass categories, during Covid-19 lockdown in Poland. Eur. J. Clin. Nutr..

[35] Błaszczyk-Bębenek E., Jagielski P., Bolesławska I., Jagielska A., Nitsch-Osuch A., Kawalec P. (2020). Nutrition behaviors in Polish adults before and during COVID-19 lockdown. Nutrients.

[36] Rodríguez-Pérez C., Molina-Montes E., Verardo V., Artacho R., García-Villanova B., Guerra-Hernández E.J., Ruíz-López M.D. (2020). Changes in dietary behaviours during the COVID-19 outbreak confinement in the Spanish COVIDiet Study. Nutrients.

[37] Faslu Rahman C.K., Sharun K., Kumar R.R., Chand S., Bardhan D., Dhama K. (2021). Impact of covid-19 pandemic and lockdown on the meat consumption pattern in India: A preliminary analysis. J. Exp. Biol. Agric. Sci..

[38] Haskaraca G., Bostanci E., Arslan Y. (2021). Effects of the COVID-19 pandemic on eating and meat consumption habits of Turkish adults. Turkish JAF Sci. Tech..

[39] Kartari A., Özen A.E., Correia A., Wen J., Kozak M. (2021). Impacts of COVID-19 on changing patterns of household food consumption: An intercultural study of three countries. Int. J. Gastron. Food Sci..

[40] Husain W., Ashkanani F. (2020). Does COVID-19 change dietary habits and lifestyle behaviours in Kuwait: A community-based cross-sectional study. Environ. Health Prev. Med..

[41] Zhao A., Li Z., Ke Y., Huo S., Ma Y., Zhang Y., Zhang J., Ren Z. (2020). Dietary diversity among Chinese residents during the COVID-19 outbreak and its associated factors. Nutrients.

[42] Revoredo-Giha C., Russo C. (2021). Purchases of meats and fish in Great Britain during the COVID-19 lockdown period. Front. Nutr..

[43] Nicola M., Alsafi Z., Sohrabi C., Kerwan A., Al-Jabir A., Iosifidis C., Agha M., Agha R. (2020). The socio-economic implications of the coronavirus pandemic (COVID-19): A review. Int. J. Surg..

[44] Levin A.T., Hanage W.P., Owusu-Boaitey N., Cochran K.B., Walsh S.P., Meyerowitz-Katz G. (2020). Assessing the age specificity of infection fatality rates for COVID-19: Systematic review, meta-analysis, and public policy implications. Eur. J. Epidemiol..

[45] Gloster A.T., Lamnisos D., Lubenko J., Presti G., Squatrito V., Constantinou M., Nicolaou C., Papacostas S., Aydın G., Chong Y.Y. (2020). Impact of COVID-19 pandemic on mental health: An international study. PLoS ONE.

[46] Muscogiuri G., Barrea L., Savastano S., Colao A. (2020). Nutritional recommendations for COVID-19 quarantine. Eur. J. Clin. Nutr..

[47] Morris L., Bhatnagar D. (2016). The Mediterranean diet. Curr. Opin. Lipidol..

[48] Widmer R.J., Flammer A.J., Lerman L.O., Lerman A. (2015). The Mediterranean diet, its components, and cardiovascular disease. Am. J. Med..

[49] Di Renzo L., Gualtieri P., Pivari F., Soldati L., Attinà A., Cinelli G., Leggeri C., Caparello G., Barrea L., Scerbo F. (2020). Eating habits and lifestyle changes during COVID-19 lockdown: An Italian survey. J. Transl. Med..

[50] Ruotolo M., Gagliardi M., Ciacci C., Zingone F., de Santis Ciacci C., Santonicola A., D’Arcangelo G., Siniscalchi M. (2021). Increased COVID-19 lockdown burden in Italian adults with gastrointestinal diseases. Nutrients.

[51] Pujia R., Ferro Y., Maurotti S., Khoory J., Gazzaruso C., Pujia A., Montalcini T., Mazza E. (2021). The effects of COVID-19 on the eating habits of children and adolescents in Italy: A pilot survey study. Nutrients.

[52] Bakhsh M.A., Khawandanah J., Naaman R.K., Alashmali S. (2021). The impact of COVID-19 quarantine on dietary habits and physical activity in Saudi Arabia: A cross-sectional study. BMC Public Health.

